# Ethanol Reduction in Montepulciano Wine: *Starmerella bombicola* Sequential Fermentation at Pilot Scale Under Aeration Conditions

**DOI:** 10.3390/foods14040618

**Published:** 2025-02-13

**Authors:** Laura Canonico, Silvia Gattucci, Laura Moretti, Alice Agarbati, Francesca Comitini, Maurizio Ciani

**Affiliations:** Department of Life and Environmental Sciences, Polytechnic University of Marche, Via Brecce Bianche, 60131 Ancona, Italy; l.canonico@univpm.it (L.C.); s.gattucci@staff.univpm.it (S.G.); laura.moretti@pm.univpm.it (L.M.); a.agarbati@univpm.it (A.A.); f.comitini@univpm.it (F.C.)

**Keywords:** yeast interactions, alcohol content, red winemaking, oxygen, *Starmerella bombicola*

## Abstract

One of the most relevant challenges in winemaking is the increase in the alcohol content of wine, mainly due to climate change. The use of selected non-*Saccharomyces* yeasts in sequential fermentation with *Saccharomyces cerevisiae* is one of the effective strategies for dealing with this issue, even if it has been poorly confirmed at the winery level. This work evaluated the use of *Starmerella bombicola* and commercial *S. cerevisiae* strains in sequential fermentation at pilot scale in winery conditions to reduce the ethanol content and obtain a wine with enhanced aroma complexity. The results showed that the sequential *S. bombicola*/*S. cerevisiae* fermentation in aeration conditions (20 mL/L/min for the first three days) resulted in a reduction in ethanol of 0.80% (*v*/*v*) compared to pure *S. cerevisiae* fermentation. The aeration conditions of sequential fermentation did not affect the fermentation performance of yeasts. The winery conditions determined, in the sequential fermentation modalities, an enhancement of wild yeasts’ presence. At the same time, the inoculation of *S. bombicola* determined an enhancement of glycerol and lactic acid, which positively influences the structure and body of the wine as well as specific aromatic notes. In winery conditions, better control of fermentation is needed to achieve potential ethanol reduction and favorable by-product formation using *S. bombicola.*

## 1. Introduction

Climate change has influenced the winemaking industry from viticultural practices to fermentation processes. Indeed, grapes have been characterized by an increase in sugar content that has led to an increase in wine’s ethanol content. In the last two decades, there has been a generalized increase of 2% (*v*/*v*) of ethanol in wines over the world [1,2]. The high ethanol content in wine can impact wine quality by increasing the perception of heat, body, viscosity, and, to a lesser extent, sweetness and acidity, and it is also related to the health aspect [1,3,4,5,6,7]. In this context, the wine sector has been trying to develop processes that produce wines with lower alcohol content without compromising their quality [8,9]. Most recently, the EU introduced the categories of “dealcoholized wine”, including wines with “actual alcoholic strength no more than 0.5% *v*/*v* ethanol”, and “partially dealcoholized wine”, where “actual alcoholic strength above 0.5% *v*/*v* ethanol is below the minimum actual alcoholic strength of the wine category” [6]. Several approaches could be used to reduce the ethanol content in wine, such as vineyard management and winemaking practices including microbiological, physical, and biochemical approaches [10,11,12,13,14]. Among the biological approaches, in addition to the use of genetically modified *Saccharomyces cerevisiae* strains, the use of non-*Saccharomyces* strains has been proposed [15,16,17]. Several non-*Saccharomyces* yeast species used in combination with *S. cerevisiae* under different fermentation conditions have formed wines with reduced ethanol concentrations [15,18,19,20,21,22,23].

Several non-*Saccharomyces* yeast species are characterized by respiro-fermentative regulatory mechanisms (Crabtree negative effect) that allow the respiration in high sugar content substrates to be carried out differently from *S. cerevisiae*, a feature that could be used to reduce ethanol content in wine [19,23,24,25,26]. In some non-*Saccharomyces* yeast species/strains, the diversion of alcoholic fermentation with an abundant formation of secondary compounds may partly explain the low ethanol yield [21,27]. Both these behaviors may contribute to achieving ethanol reduction [28]. Several non-*Saccharomyces* yeast strains were investigated under aerated or other fermentation conditions with the following ethanol reduction levels: *Metschnikowia pulcherrima* (0.8–1.6%); *Starmerella bombicola* (0.6–1.6%); *Torulaspora delbrueckii* (0.3–1.5%); *Zygosaccharomyces bailii* (1–2%); *Starmerella bacillaris* (0.3–0.8%); *Schizosaccharomyces pombe* (0.4–0.65%); *Schizosaccharomyces japonicus* (1.7–2.4%); *Lachancea thermotolerans* (0.4–1.2%); *Hanseniaspora opuntiae* (0.6–1.3%); and *Hanseniaspora osmophila* (0.8–1.3%), which can decrease ethanol yields through respiration [29,30,31,32,33]. Among them, *S. bombicola* (formerly *Candida stellata*) showed promising applications in winemaking for its by-products, particularly glycerol [30,34]. Following the results of previous works [29,30], here, *S. bombicola*/*S. cerevisiae* sequential fermentation was evaluated under winery conditions at the pilot scale under aeration (20 mL/L/min for the first three days) for ethanol reduction and the overall improvement of the wine profile. The influence of cellar conditions and the red winemaking process on the use of *S. bombicola* for ethanol reduction was also evaluated.

## 2. Materials and Methods

### 2.1. Yeast Strains

The non-*Saccharomyces* yeast strain used in this study was *S. bombicola* DiSVA66 (DBVPG # 3827 Industrial Yeast Collection of the University of Perugia). This strain was previously selected and used in laboratory-scale sequential fermentation for ethanol reduction in different fermentation conditions [15,30].

*S. cerevisiae* commercial strain Lalvin EC1118 (Lallemand Inc., Toulouse, France) was used as a control strain and in sequential fermentation with *S. bombicola.* All the strains were maintained at −80 °C for long-term storage in cryovials supplemented with 40% (*w*/*v*) glycerol as the cryoprotective agent. Subsequently, the strains were cultured on Yeast Peptone Dextrose (YPD) agar medium at 25 °C for 48–72 h and stored at 4 °C.

### 2.2. Pilot Scale Fermentation

Montepulciano grape juice, a red grape variety provided by the winery Terre Cortesi Moncaro s.r.l., Montecarotto (AN), Italy, was used at pilot scale fermentation. Montepulciano grape juice showed the following characteristics: pH 3.52, sugar content 255.71 g/L, malic acid 0.87 g/L, lactic acid 0.36 g/L, total acidity 4.02 g/L. Modified YPD (yeast extract 0.5%, peptone 0.1%, glucose 2%) was used to obtain biomass for fermentation trials. *S. bombicola* was incubated at 25 °C for 72 h under shaking conditions (150 rpm). Biomass was harvested by centrifugation. Cell concentration was determined through the Thoma-Zeiss Counting Chamber. The fermentation trials were carried out in duplicate in 150 L steel tanks containing 100 L. A uniform batch of Montepulciano grapes (500 Kg) was destemmed and pressed, and grape must with skins was distributed into four steel tanks. Sequential trials were inoculated with *S. bombicola* DiSVA66 at a concentration of 5 × 10^6^ cells/smL at 25 °C. A supplement of aeration was maintained using 20 mL/L/min of airflow during the initial 72 h. After this, no aeration was applied, and *S. cerevisiae* was inoculated (1 × 10^6^ cells/mL). Pure fermentation trials of *S. cerevisiae* (inoculum 1 × 10^6^ cells/mL) were used as a control. The skins were kept in both fermentation trials until the 0 °Babo degree (about 20 g/L of reduced sugars), and the fermentation processes were conducted without skin. The temperature was maintained at 25 ± 1 °C.

### 2.3. Biomass and Sugar Kinetics

Biomass evolution was evaluated by viable cell count (CFU/mL) on lysine agar selective medium and WL nutrient agar (Oxoid, Hampshire, UK). Wild non-*Saccharomyces* yeasts (WNSs) were easily distinguished by *S. bombicola* through macro- and microscopic characterization of the colony on WL nutrient agar. To confirm the belonging of the isolates to the species *S. bombicola*, some strains underwent DNA extraction and were then identified through ITS1- 5.8S rRNA-ITS2 region analyses using the primer pairs ITS1 (5′-TCCGTAGGT GAACCTCGCG-3′)-ITS4 (5′-TCCTCCGCTTTATTG ATATGC-3′). PCR products were separated by horizontal electrophoresis (Bio-Rad, Hercules, CA, USA) in a 1.5% (*w*/*v*) agarose gel using 0.5×TBE buffer and used for identification by sequencing [35]. The genomic sequences obtained were compared with those already present in the data library using the BLAST program and the GenBank database of the ITS. The fermentations were carried out in duplicate. The glucose and fructose (K-FRUGL) concentrations were determined using specific enzyme kits (Megazyme International, Wicklow, Ireland).

### 2.4. Analytical Determinations

Total acidity [36], organic acids [37], volatile acidity [38], pH [39], ethanol content [40], sugar content [41], and free and total SO_2_ [42] were evaluated according to the use of the standard methods of OIV. Acetaldehyde, ethyl acetate, and higher alcohols were analyzed using a gas chromatograph system (GC-2014; Shimadzu, Kyoto, Japan) using direct injection [15]. Samples were injected into a 30 m × 0.32 mm column with a 0.25 μm film thickness (Zebron ZB-WAXPlus; Phenomenex, Torrance, CA, USA) using 1-pentanol (162 mg/L) as an internal standard. Helium served as carrier gas. A Shimadzu gas chromatograph (Japan) equipped with a flame ionization detector was used. The oven temperature ranged from 40 °C to 200 °C. The oven temperature ranged from 40 °C for 5 min, then 5 °C/min until 200 °C for 10 min, while the injector and detector temperatures were maintained at 220 °C. The main volatile compounds were analyzed using the solid-phase microextraction (HS-SPME) method. Five mL of each sample was placed in a vial containing 1 g NaCl closed with a septum-type cap. HS-SPME was carried out under magnetic stirring for 10 min at 25 °C. After this period, an amount of 3-octanol as the internal standard (1.6 mg/L) was added, and the solution was heated to 40 °C and extracted with a Divinylbenzene/Carboxen/Polydimethylsiloxane (DVB/CAR/PDMS) fiber (Sigma-Aldrich, St. Louis, MI, USA) for 30 min by insertion into the vial headspace. The compounds were desorbed by inserting the fiber into a Shimadzu gas chromatograph GC injector for 5 min. The following glass capillary column was used: 0.25 μm Supelcowax 10 (length, 60 m; internal diameter, 0.32 mm). The fiber was inserted in split–splitless mode. The compounds were identified and quantified by comparisons with calibration curves for each compound.

### 2.5. Sensorial Analysis

At the end of the fermentation, the wines were decanted and after three months, transferred into filled 750 mL bottles, closed with the crown cap, and maintained at 4 °C until sensory analysis. After this period of refinement, they were subjected to sensory evaluation. A group of 10 testers, 8 males and 2 females aged 25–45 years (80% expert and 20% non-expert), used a score scale of 1 to 10, where 10 was the score that quantitatively represented the best judgment (maximum satisfaction), and 1 was the score to be attributed in case of poor satisfaction. The expert testers were composed of oenologists, sommeliers, and wine producers. The order of presentation was randomized among judges. A list of descriptors related to both the olfactory aromatic notes (ripe fruit, tropical fruit, citrus, honey, spicy, aromatic herbs, herbal, and floral) and the taste features (acidity, bitter, softness, structure, balance, tannicity, and intensity). Their data were combined, and the means were subjected to statistical analysis. The data processed in this way were used to provide information on both the contributions of each descriptor to the overall organoleptic quality of the wines and the significant differences between the wines about each descriptor.

### 2.6. Statistical Analysis

Analysis of variance (ANOVA) was applied to the experimental data for the main enological characteristics and volatile compounds of the wines. The data were analyzed using STATISTICA 7—version 7, the statistical software. Duncan tests were used to detect significant differences, where significance was associated with *p*-values < 0.05. The data from the sensory analysis were also subjected to Fisher ANOVA to determine the significant differences (*p* < 0.05).

## 3. Results

### 3.1. Fermentation Kinetics and Biomass Evolution

**Figure 1:**
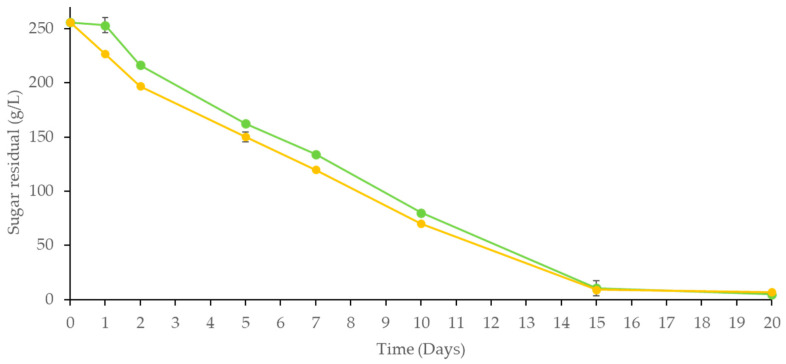
Kinetics of sugar consumption of pure and sequential fermentation are carried out at an industrial level. *S. bombicola*/*S. cerevisiae* (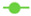), *S. cerevisiae* pure culture (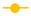).

The kinetics of sugar consumption, reported in Figure 1, showed a similar trend among the two fermentation trials. It should be highlighted that the *S. bombicola*/*S. cerevisiae* sequential fermentations in aeration conditions exhibited higher fermentation kinetics up to the tenth day of fermentation (especially on the third day of fermentation) and then overlapped to *S. cerevisiae* pure culture.

**Figure 2:**
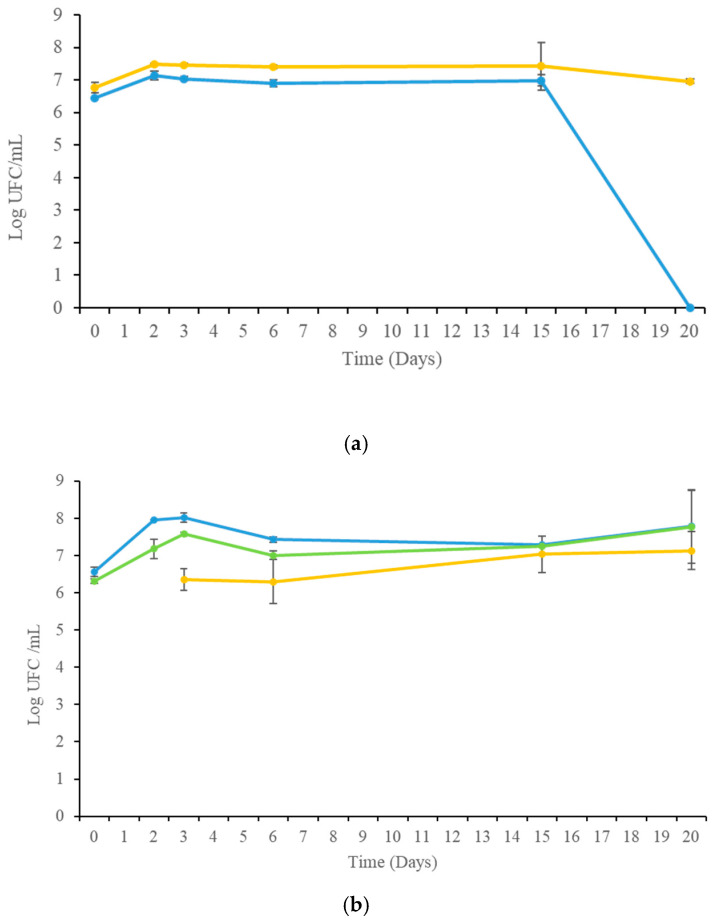
Growth kinetics of *S. cerevisiae* pure culture in Montepulciano grape juice (**a**) and *S. bombicola*/*S. cerevisiae* sequential fermentation (**b**). *S. cerevisiae* (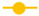), wild yeasts (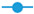), *S. bombicola* (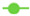). Wild yeasts were apiculate yeasts (*Hanseniaspora* spp.), *Starmerella baciillaris*, and occasionally (0th and 2nd day) *Metschnikowia* spp. and *Pichia* spp.

The biomass evolution of fermentations is reported in Figure 2. *S. cerevisiae* pure culture (Figure 2a) reached the maximum biomass concentration (over 10^7^ CFU/mL) on the third day of fermentation and then remained constant until the end of the fermentation process. The same trend was exhibited by wild non-*Saccharomyces* (mainly represented by apiculate yeasts and *Starmerella baciillaris* and occasionally by *Metschnikowia* spp. and *Pichia* spp.) present in the initial grape juice until the fifteenth day of fermentation and then disappeared. *S. bombicola* in sequential fermentation (Figure 2b) achieved the highest biomass concentration on the third day of fermentation and remained constant until the end of fermentation. A similar trend was exhibited by wild non-*Saccharomyces* yeasts, showing, however, a slightly higher concentration. Regarding *S. cerevisiae* inoculated on the third day of fermentation, it showed a limited evolution until the end of fermentation. The high presence of wild non-*Saccharomyces* yeasts may be due to the practice of the red winemaking process that requires the presence of grape skins during fermentation.

### 3.2. Main Fermentation Parameters

Table 1 reports the data of the main analytical characters on the third day of fermentation. The only significant difference was in the ethanol content: sequential fermentation *S. bombicola*/*S. cerevisiae* showed lower ethanol (1.44% *v*/*v*) compared to *S. cerevisiae* pure culture (3.24% *v*/*v*).

The low value of the ethanol yield in the first three days of fermentation highlighted the reduced ethanol produced by sequential fermentation trials due to the inoculum of *S. bombicola* and wild yeasts’ presence as well as air supplementation.

The data of the main analytical characters of the final wines are reported in Table 2. *S. bombicola* sequential fermentation with *S. cerevisiae* led to wine with a significant reduction in ethanol (0.8% *v*/*v*) and total SO_2_ and an increase in glycerol compared with *S. cerevisiae* pure culture. No significant differences were shown for the other parameters analyzed, even if an increase in volatile acidity was detected without determining relevant influence on its perception. This behavior may be due to the presence and development of wild yeast during the fermentation process in sequential aeration conditions.

### 3.3. The Main Volatile Compounds

The data of the main volatile compounds of *S. cerevisiae* pure cultures and sequential fermentation are reported in Table 3. Regarding ester compounds, *S. bombicola* sequential fermentation led to wine with a significant increase in ethyl acetate and phenyl ethyl acetate, which are responsible for fruity, floral aromas, and the wine was sweeter in comparison to that of the *S. cerevisiae* pure culture. Regarding the other esters, no significant differences were shown among the compounds analyzed. Moreover, the sequential fermentation significantly increased the linalool and isobutanol concentrations compared to the *S. cerevisiae* pure culture. On the other hand, *S. cerevisiae* starter strains exhibited significant increases in nerol and amyl alcohol. No significant differences were shown for the other volatile compounds tested.

### 3.4. Sensorial Analysis

**Figure 3:**
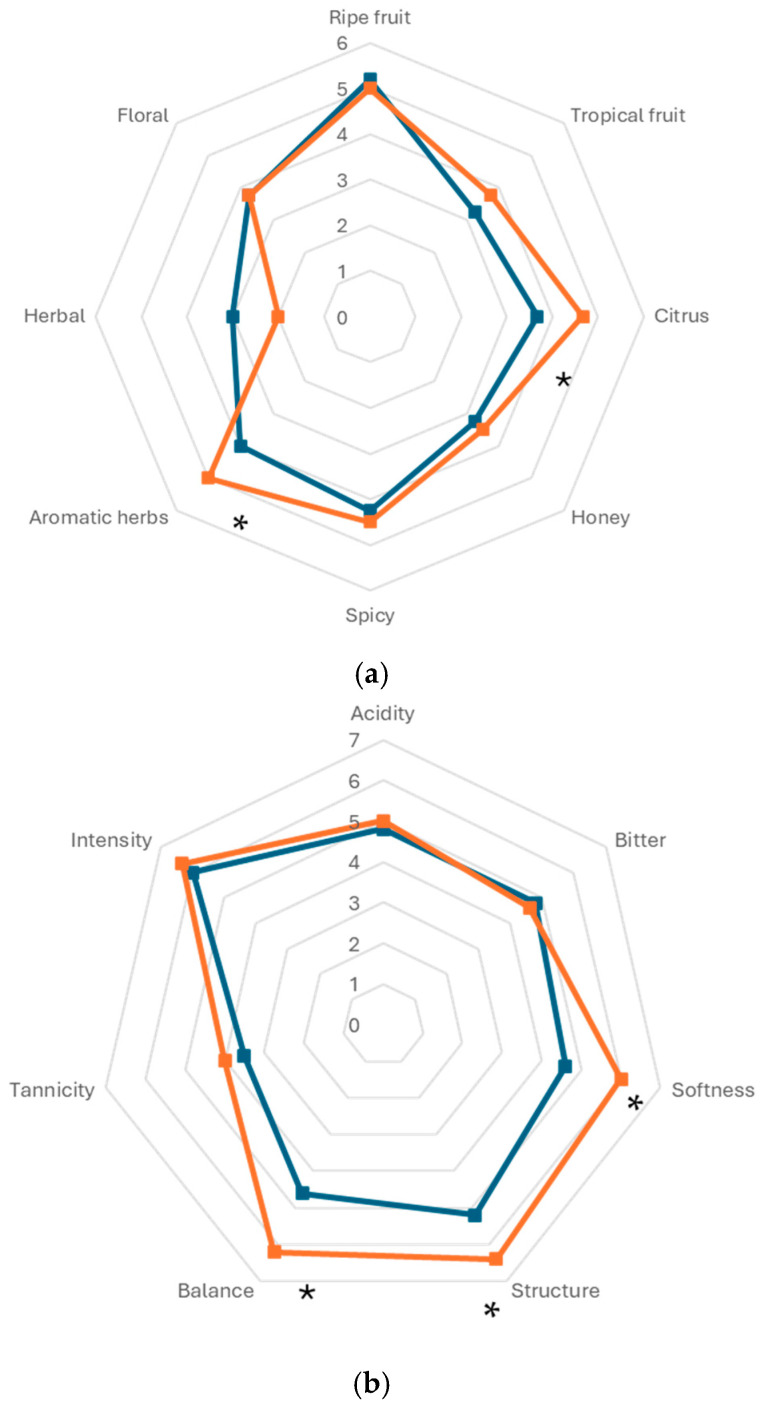
Sensory analysis of wines produced in the pure and mixed fermentations: (**a**) olfactory analysis and (**b**) taste analysis. *S. cerevisiae* (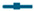), *S. bombicola*/*S. cerevisiae* (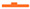). *, Significantly different (Fisher ANOVA; *p*-value 0.05).

The wines underwent sensory analysis, and the data reported in Figure 3 showed significant differences for all the wines analyzed for some of the aromatic notes. In particular, the olfactory analysis highlighted a significant increase in aromatic herbs and citrus notes in wine carried out with *S. bombicola*. Moreover, in regard to taste analysis, the same wines showed a significant increase in balance, structure, and softness. In general, the results highlighted the positive judgment of testers regarding each wine, characterized by specific aromatic notes and without defects.

## 4. Discussion

In the last two decades, there has been an average increase of two degrees of alcohol in wine due to climate change and the technology used and required by consumer demands. Overall, the combination of health, economic, and quality issues associated with high-alcohol wines has led to significant interest in the development of technologies for producing wines with reduced ethanol concentrations. Several approaches have been proposed to reduce the ethanol content in wine. Among biotechnological approaches, the use of non-*Saccharomyces* yeasts in different fermentation conditions can lead to the achievement of this goal [8,15,18,19,23,43,44,45]. A valuable fermentation strategy is the addition of air during the first stages of fermentation to allow the consumption of sugar through yeast respiration [46,47]. Among non-*Saccharomyces* yeasts, *S. bombicola* has been extensively studied in different fermentation conditions and grape varieties to achieve the goal of improving some specific analytical compounds [48,49].

In this investigation, the use of *S. bombicola*/*S. cerevisiae* under limited aeration conditions (20 mL/L min) at pilot scale sequential fermentation was evaluated to reduce the ethanol content and increase the aroma complexity in Montepulciano wine production. The use of selected non-*Saccharomyces* yeasts in sequential fermentation with *S. cerevisiae* to reduce the ethanol content was well investigated at the laboratory level but is poorly evaluated under winery conditions [43]. In previous studies, this strain of *S. bombicola* was used in different fermentation conditions to reduce the alcohol content in wine and increase the glycerol content [15,48,49]. *S. bombicola* was used in immobilized form to start fermentation, followed by inoculation of free *S. cerevisiae* cells. This led to wine with a 1.6% *v*/*v* reduction in the final ethanol content in comparison with *S. cerevisiae* starter strains [15]. In a subsequent study, *S. bombicola* bench-top fermentation conducted in Verdicchio grape juice supplemented with 20 mL/L/min of air during the first 72 h produced a wine with an ethanol reduction of 1.46% (*v*/*v*) [30]. Here, under winery conditions, *S. bombicola*/*S. cerevisiae* red winemaking fermentation, supplemented with air during the first 72 h, resulted in a reduction of ethanol of 0.80% (*v*/*v*). In comparison with the laboratory conditions and immobilized cell inoculum, there was a lower ethanol reduction, even if an 0.80% (*v*/*v*) reduction may be a valuable starting point in winery conditions. The scale-up conditions showed several concerns linked to winery conditions and difficulty in controlling the fermentation process with particular reference to mixed fermentations. Also, in this case, a relevant presence of wild yeasts (mainly *H. uvarum* and *S. bacillaris*) in the sequential trials was found. The presence of grape skins (fermentation with maceration) facilitates the presence of wild yeasts in the must and makes it more difficult for the inoculated *S. bombicola* strain to dominate the fermentation process. Better control of wild yeasts and an increase in the inoculation level of *S. bombicola* could enhance the competitivity. Regarding the structure and aromatic profile of wine, this fermentation strategy led to an increase in glycerol content of approximately 4.5 g/L. The ethanol reduction achieved in the present work could be, at least in part, explained by the relevant increase in glycerol, as previously reported by Ciani and Ferraro [50]. A similar result was obtained with *Candida zemplinina* (synonym *Starmerella bacillaris*, a closely related species with similar oenological features to *S. bombicola*), which was widely investigated to produce wine with fewer ethanol levels and higher glycerol content [51]. Oxygen supplementation influences the formation of some volatile compounds such as esters, higher alcohols, ethyl esters, and acetate esters [52,53,54,55,56]. In the present study, the air supplementation increased ethyl acetate and phenyl ethyl acetate in *S. bombicola*/*S. cerevisiae* trials in regard to the ester compounds and only isobutanol in regard to higher alcohols. Sensory analysis confirmed the analytical profile, highlighting citrus notes, balance, structure, and softness in inoculated wines with *S. bombicola* and determining the preference of the tasting panel. In summary, our results confirm the ability of the *S. bombicola* selected strain in sequential fermentation under aeration conditions to reduce the ethanol content in wine and increase the glycerol content in pilot scale winery conditions. Moreover, *S. bombicola* significantly influenced the aroma composition of wine, leading to a more balanced wine with lower ethanol concentration and a pleasant sensory profile.

## 5. Conclusions

For the first time, a sequential fermentation of *S. bombicola*/*S. cerevisiae* in partial aeration conditions (three days) was conducted in a winery at the pilot scale level. The results previously obtained at the laboratory scale were substantially confirmed. Here, an ethanol reduction of close to 1% and an enhancement of glycerol of 50% were obtained. The analytical profile and sensory evaluation of the wines revealed that *S. bombicola*/*S. cerevisiae* sequential fermentation displayed better overall characteristics than pure fermentation with *S. cerevisiae.* On the other hand, the relevant presence and development of wild yeasts in the inoculum of *S. bombicola* indicates that better control of the fermentation process is necessary. The results indicated that this biotechnological strategy could be used in favorable vintages where the control of wild yeasts is easier. Further studies should be carried out to confirm and improve these results under winery conditions through the enhancement of the starter inoculum and better control of the wild microbiota.

## Figures and Tables

**Table 1:** Chemical characterization of wine on the third day of fermentation. Data are means ± standard deviations. Values displaying different superscript letters (^a,b^) within each column are significantly different according to Duncan’s tests (*p* < 0.05).

Sample	Ethanol (% *v*/*v*)	Ethanol Yield (wt/vol%)	Total Acidity (as Tartaric Acid g/L)	pH	Volatile Acidity (as Acetic Acid g/L)	Sugar Content (g/L)
*S.cerevisiae* pure culture	3.24 ± 0.03 ^a^	0.54 ± 0.04 ^a^	5.92 ± 0.06 ^a^	3.50 ± 0.02 ^a^	0.21 ± 0.01 ^a^	196.01 ± 1.96 ^a^
*S. bombicola*/*S. cerevisiae*	1.77 ± 0.14 ^b^	0.45 ± 0.10 ^b^	5.69 ± 0.17 ^a^	3.50 ± 0.01 ^a^	0.25 ± 0.05 ^a^	216.40 ± 7.21 ^a^

**Table 2:** Chemical characterization of resulting wine. Data are means ± standard deviations. Values displaying different superscript letters (^a,b^) within each column are significantly different according to Duncan’s tests (*p* < 0.05).

Sample	Ethanol (% *v*/*v*)	Ethanol Yield (wt/vol%)	Total Acidity (as Tartaric Acid g/L)	pH	Volatile Acidity (as Acetic Acid g/L)	Free SO_2_	Total SO_2_	Sugar Content (g/L)	Net Extract (g/L)	Glycerol (g/L)
*S. cerevisiae* pure culture	15.18 ± 0.02 ^b^	0.59 ± 0.02 ^b^	6.89 ± 0.03 ^a^	3.52 ± 0.01 ^a^	0.33 ± 0.01 ^a^	12.50 ± 0.71 ^a^	41.50 ± 2.12 ^a^	2.4 ± 0.1 ^a^	27.25 ± 0.01 ^a^	8.98 ± 0.85 ^b^
*S. bombicola*/ *S. cerevisiae*	14.39 ± 0.25 ^a^	0.56 ± 0.01 ^a^	5.94 ± 0.97 ^a^	3.68 ± 0.10 ^a^	0.60 ± 0.16 ^a^	10.00 ± 1.41 ^a^	28.50 ± 0.71 ^b^	2.9 ± 0.2 ^a^	30.89 ± 0.33 ^a^	13.60 ± 0.36 ^a^

**Table 3:** The main volatile compounds (mg/L) of *S. cerevisiae* pure culture and *S. bombicola* sequential fermentations. OAV = odor activity value. Data are means ± SD from two independent experiments and two repetitions of analysis. Data with different superscript letters (^a,b^) within each column are different according to Duncan’s tests (0.05%).

ESTERS	*S. cerevisiae* Pure Culture	OAV	*S. bombicola*/*S.* *cerevisiae*	OAV
Ethyl butyrate	1.28 ± 0.72 ^a^	3.2	3.71 ± 0.94 ^a^	9.28
Ethyl acetate	23.70 ± 4.46 ^b^	1.98	95.21 ± 3.07 ^a^	7.93
Phenyl ethyl acetate	0.07 ± 0.01 ^b^	0.96	0.09 ± 0.00 ^a^	1.23
Ethyl octanoate	0.003 ± 0.001 ^a^	0.01	0.005 ± 0.001 ^a^	0.01
Isoamyl acetate	0.56 ± 0.06 ^a^	3.5	0.66 ± 0.05 ^a^	4.13
Hexyl acetate	0.002 ± 0.000 ^a^	0.00	0.004 ± 0.00 ^a^	0.01
Diethyl succinate	0.02 ± 0.01 ^a^	0.00	0.07 ± 0.02 ^a^	0.00
CARBONYL COMPOUNDS				
Acetaldehyde	1.85 ± 0.67 ^a^	3.7	20.59 ± 10.68 ^a^	41.18
MONOTERPENS				
Linalool	0.03 ± 0.01 ^b^	1.20	0.13 ± 0.01 ^a^	5.20
Geraniol	0.02 ± 0.00 ^a^	0.67	0.02 ± 0.00 ^a^	0.67
Nerol	0.04 ± 0.00 ^a^	2.67	0.02 ± 0.01 ^b^	1.33
NORISOPRENOIDS				
β-damascenone	0.02 ± 0.01 ^a^	0.00	0.03 ± 0.01 ^a^	0.00
HIGHER ALCOHOLS				
Hexanol	0.02 ± 0.00 ^a^	0.00	0.02 ± 0.00 ^a^	0.00
β-Phenyl ethanol	96.2 ± 1.12 ^a^	0.69	113.7 ± 0.68 ^a^	0.81
n-propanol	18.70 ± 1.80 ^a^	0.06	17.26 ± 1.36 ^a^	0.06
Isobutanol	16.41 ± 0.95 ^b^	0.41	45.93 ± 4.79 ^a^	1.15
Amyl alcohol	71.08 ± 0.58 ^a^	1.11	44.00 ± 4.20 ^b^	0.69
Isoamyl alcohol	158.97 ± 1.04 ^a^	2.65	141.00 ± 11.48 ^a^	2.35

## Data Availability

The original contributions presented in the study are included in the article, further inquiries can be directed to the corresponding author.
